# DNA methylation is critical for tooth agenesis: implications for sporadic non-syndromic anodontia and hypodontia

**DOI:** 10.1038/srep19162

**Published:** 2016-01-13

**Authors:** Jing Wang, Ke Sun, Yun Shen, Yuanzhi Xu, Jing Xie, Renhuan Huang, Yiming Zhang, Chenyuan Xu, Xu Zhang, Raorao Wang, Yunfeng Lin

**Affiliations:** 1 Department of Stomatology, Shanghai Tenth People’s Hospital, Tongji University School of Medicine, No.301, Middle Yanchang Road, Shanghai 200072, P.R. China; 2 State Key Laboratory of Oral Diseases, West China Hospital of Stomatology, Sichuan University, No.14., 3rd Sec, Ren Min Nan Road, Chengdu 610041, P.R. China

## Abstract

Hypodontia is caused by interactions among genetic, epigenetic, and environmental factors during tooth development, but the actual mechanism is unknown. DNA methylation now appears to play a significant role in abnormal developments, flawed phenotypes, and acquired diseases. Methylated DNA immunoprecipitation (MeDIP) has been developed as a new method of scanning large-scale DNA-methylation profiles within particular regions or in the entire genome. Here, we performed a genome-wide scan of paired DNA samples obtained from 4 patients lacking two mandibular incisors and 4 healthy controls with normal dentition. We scanned another female with non-syndromic anodontia and her younger brother with the same gene mutations of the PAX9,MSX1,AXIN2 and EDA, but without developmental abnormalities in the dentition. Results showed significant differences in the methylation level of the whole genome between the hypodontia and the normal groups. Nine genes were spotted, some of which have not been associated with dental development; these genes were related mainly to the development of cartilage, bone, teeth, and neural transduction, which implied a potential gene cascade network in hypodontia at the methylation level. This pilot study reveals the critical role of DNA methylation in hypodontia and might provide insights into developmental biology and the pathobiology of acquired diseases.

Odontogenesis is a complex process involving multiple and overlapping molecular events in signaling pathways between the epithelium and neural-crest-derived mesenchyme^1^,^2^,^3^. Environmental factors (irradiation, chemotherapy, drugs, etc.) or gene mutation in any stage of the process can affect or stop tooth development, resulting in tooth agenesis, which consists of abnormalities in tooth number, shape, size, and structure^4^,^5^. Hypodontia presents heterogeneous phenotypes ranging from a single congenitally missing tooth to more than 6 teeth (oligodontia), excluding third molars and the complete absence of teeth (anodontia). Hypodontia occurs either as an isolated non-syndromic trait or as part of a syndrome^5^. In most individuals with hypodontia, only one or two teeth are affected^5^,^6^.

However, the etiology of tooth agenesis remains to be elucidated. With increasing understanding of the genetic progress of dental development, over 200 genes have been identified as potential candidate genes for hypodontia^7^,^8^. Among them, MSX1 (MIM# 142983), PAX9 (MIM# 167416), AXIN2 (MIM# 604025), WNT10A (MIM# 606268), and EDA (MIM# 300451) have been reported to be responsible for isolated/non-syndromic hypodontia^4^,^5^,^9^,^10^,^11^,^12^,^13^ in mouse and (partially) human models.

Interestingly, our previous study^14^ reported on a non-syndromic anodontia female proband and her younger brother, who had a normal phenotype. A pedigree analysis was performed, and the two siblings were found to share the same variations in important tooth-agenesis-related genes (PAX9 and AXIN2). Although we could not exclude the possibility that other unknown gene mutations led to the different phenotype between the two siblings (only MSX1, PAX9, AXIN2, and EDA were analyzed), it may be considered that the non-gene regulation is a causative factor. Similarly, three large-scale clinical studies of monozygotic twin (MZT) pairs also presented discordant phenotypes, including the number of congenitally missing or supernumerary teeth^15^. Townsend and co-workers^16^,^17^,^18^,^19^ proposed that epigenetic factors may explain the considerable differentiated expression of supernumerary or missing teeth in a pair of MZTs. These phenomena inspired us to investigate the contributions and interactions of genetic, environmental, and epigenetic influences on tooth agenesis.

DNA methylation and histone modification are two of the most common epigenetic alteration activities, namely the covalent modifications of DNA^20^. It affect chromatin inactivation, specific gene expression related to embryonic growth, cell differentiation, and cancer progression, through spatial arrangement of cells and the timing of interactive signaling^21^,^22^. For example, it has been found that histone demethylase regulates dental stem cell differentiation^23^. In response to mineralization, H3K27me3-mediated repression of DSPP and dentin matrix protein 1 genes are expressed in dental follicle cells but not in dental pulp cells, indicating the significant role of epigenetic regulatory mechanisms in the terminal differentiation of odontogenic neural crest lineages^24^. Further, histone demethylase KDM6B has been shown to promote odontogenic differentiation of dental MSCs^25^. As regards human trials, Yin and colleagues^26^ recently reported that the methylation state of the EDA promoter was associated with X-linked hypohidrotic ectodermal dysplasia (XLHED) in a Chinese population. Eighteen (78.26%) carriers were hypermethylated at 4 sites.

However, previous studies have focused only on the methylation states of specific genomic sites, and, to the best of our knowledge, there are no published studies of genome-wide methylation status in hypodontia. The advent of methylated DNA immunoprecipitation (MeDIP), new technique to determine DNA-methylation profiling within functional regions (e.g., promoters) or in the entire genome, has made such studies feasible. This approach is based on the enrichment of methylated DNA with an antibody that specifically binds to 5-methyl-cytosine and can be combined with PCR, microarrays, or high-throughput sequencing. Details of the assay have been well-reviewed^27^. Since the introduction of MeDIP, this method has been widely used to map methylation files in different organisms^28^,^29^, to distinguish cancer^30^,^31^, to analyze metabolite diseases^32^, and to identify epigenomic changes of differentiation in embryos or stem cells^33^,^34^. Therefore, based on our previous results^14^, we recruited patients with paired matches and DNA samples were obtained^35^, to investigate the association of the methylation state of hypodontia-related genes with the phenotype. We attempted to gain more insight into the epigenetic regulation in tooth agenesis, by collecting and analyzing the differences in the genome-wide DNA methylation information of hypodontia-related genes between people with phenotypes and those without.

## Results

### Distribution of enrichment peaks (EPs) in samples from individuals with non-syndromic tooth agenesis (NSTA) and unaffected control individuals

**Figure 1:**
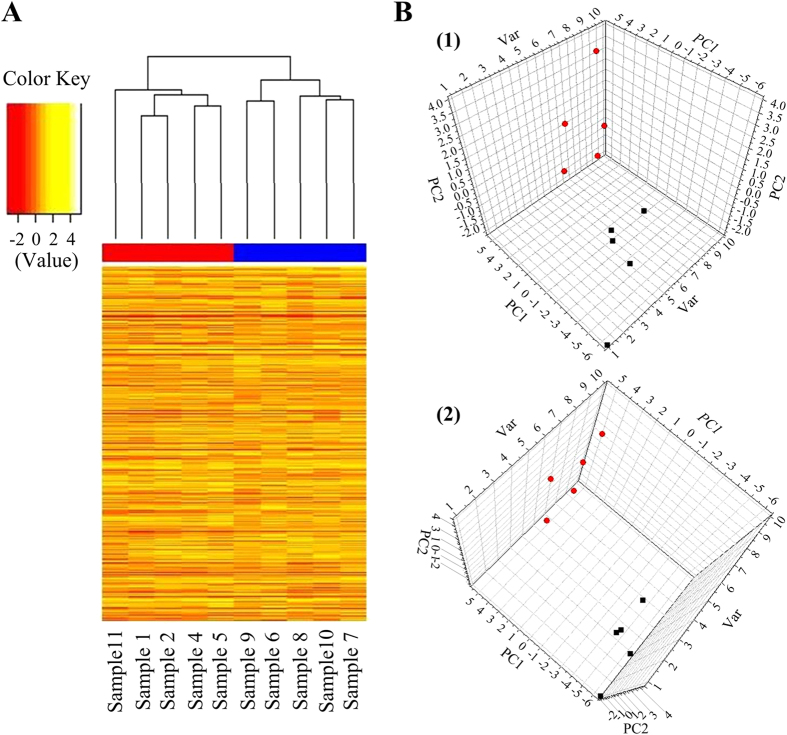
Difference of whole-gene methylation between NSTA (the red groups: 1, 2, 4, 5, 11) and control groups (the blue groups: 6, 7, 8, 9, 10). **(A**) The cluster diagram of whole genes which showed significant differences between the NSTA group and the control (10 samples) in methylation level. The differentiation ratio ranged from −2 to 4. Each row represents a gene, and each column represents a sample. There is clustering into two groups that correspond to the NSTA and the control groups. Red, hypomethylation; yellow, hypermethylation. **(B)** 3D score plot of the PCA for whole-gene methylation. The two components (PC1, PC2) and Var shown were able to cluster the NSTA group and the control samples.

**Figure 2:**
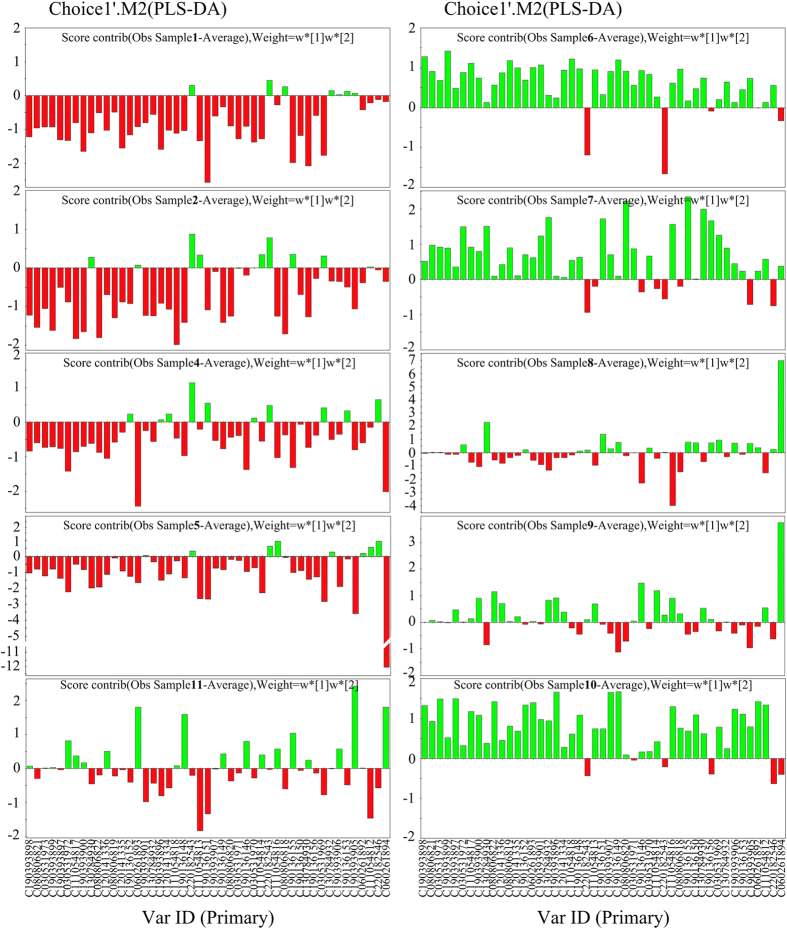
The regression coefficients of the PLS-DA model of the NSTA (sample 1) and the control (sample 6). Positive coefficients (green) indicate relatively higher values for degree of methylation in NSTA compared with the control samples, whereas negative coefficients indicate lower values (red). The magnitude of the coefficient represents the relative importance of each data bit on the separation contribution.

MeDIP permits the highly efficient enrichment of methylated DNA and can be combined with large-scale analysis using existing DNA microarrays. We identified 28,820 probes covering 6636 genes with differential methylation levels, as shown by the Differential enrichment peaks (DEP) results for whole-gene methylation (Fig. 1A). These data were analyzed by two pattern-recognition methods, principal components analysis (PCA) and partial least-squares discriminant analysis (PLS-DA), to identify the differential methylated genes responsible for phenotype differences. The results of a 3-D PCA score plot distinguished NSTA samples and control samples for PC1, PC2, and Var (Fig. 1B). Figure 2 illustrates the regression coefficients of the PLS-DA model, indicating significant differences in methylation status of particular genes between the two groups. We compared methylation sites on chr1 between the two groups (Appendix Figure 1), to illustrate the most typical results. Given that the promoters located near the transcription start site (TSS) were more significant in the regulation of gene expression^36^, we focused on the methylation state of gene promoters located in the region −3000 to +3000 bp from the TSSs in this study. This region contained approximately 1621–3314 peaks per sample. The distribution of EPs in each region was similar in patient and control samples.

### Promoter methylation profiling

To obtain a global view of the methylation profiles of the NSTA patients and the control individuals, we first corrected the MeDip data for background and normalization (bioconductor packages Ringo, limma, and MEDME). Next we analyzed candidate gene variations by comparing NSTA patients with the control individuals. Two steps were adopted to analyze the data. The inclusion criteria for both analysis strategies are shown in Appendix Figure 2.

**Figure 3:**
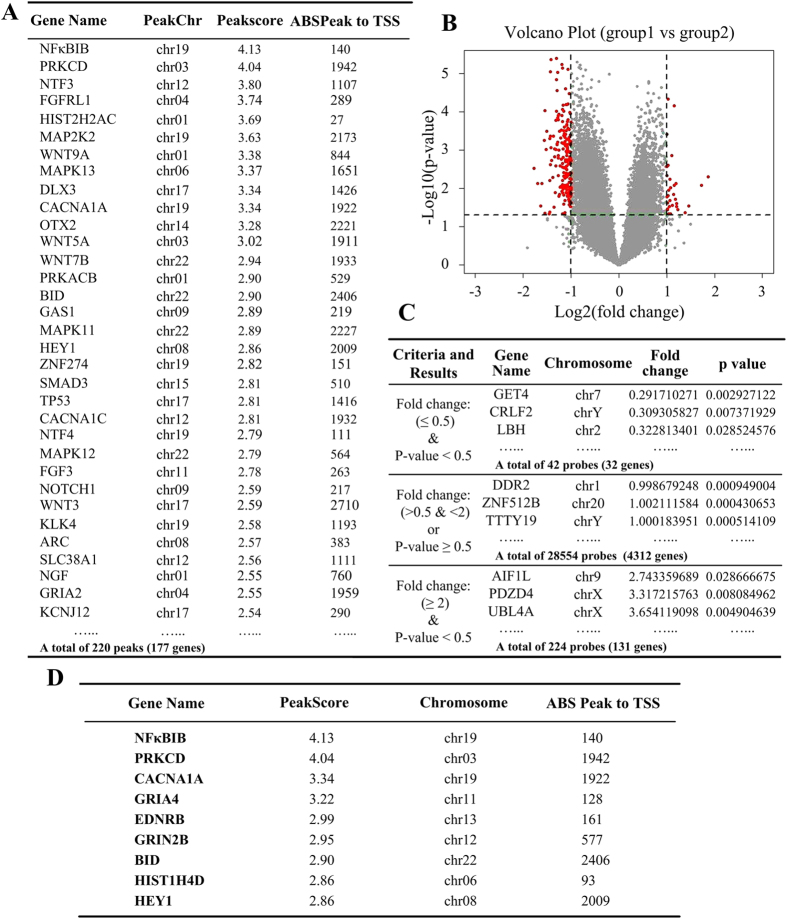
The two-step screening results of genes of interest (GOI). (**A**) The tabulated results of NimbleScan v2.5 analysis (Inclusion criteria: [1] peak score >2, *P* < 0.05; [2] distance to TSS within −3 kb to +3 kb). **(B)** Volcano plot of probe-specific analysis data demonstrates 163 genes with significantly different methylation levels (cutoff ratio >2 or < 0.5, *P* < 0.05; red, fold change ≤0.5 or >2; grey, >0.5 or <2). Log2 (fold change) is shown on the x-axis, and −log10 (*P*-value) is shown on the y-axis. **(C)** The tabulated results of probe-specific analysis. The intersection of **(A,C)** was subjected to GO and pathway analysis, and results are shown in **(D).** The tabulation reflects 9 genes of interest. Chr: chromosome.

We adopted software (NimbleScan v2.5) analysis and probe-specific analysis independently for the normalized data, to minimize false-negatives and false-positives, respectively. The results of NimbleScan v2.5 analysis demonstrated that 220 peaks presented significantly different methylation levels, as shown in Fig. 3A. Sixty-one genes were located in HCP, 22 genes in ICP, and 137 in LCP (Appendix Table 1). The primary results of probe-specific analysis revealed that 224 probes (covering 131 genes) were hypermethylated and 42 probes (covering 32 genes) were hypomethylated, respectively, as presented in Fig. 3C (Appendix Table 2). Figure 3B is the volcano plot showing the –Log *P*-values for data from probe-specific analysis (Appendix Table 2). To identify the genes of interest (GOI) for further analysis, we selected the intersection of Appendix Tables 1 and 2 . Then Gene ontology (GO), and pathway analysis was performed. We identified 9 eligible genes, namely, NFKBIB, PRKCD, CACNA1A, GRIA4, EDNRB, GRIN2B ,BID, HIST1H4D, and HEY1, as illustrated in Fig. 3D. These stringent restrictions were carried out to reduce the possibility of introducing too many false-positives and thus guaranteed the accuracy.

### Results of differential enrichment peak (DEP) cluster analysis

**Figure 4:**
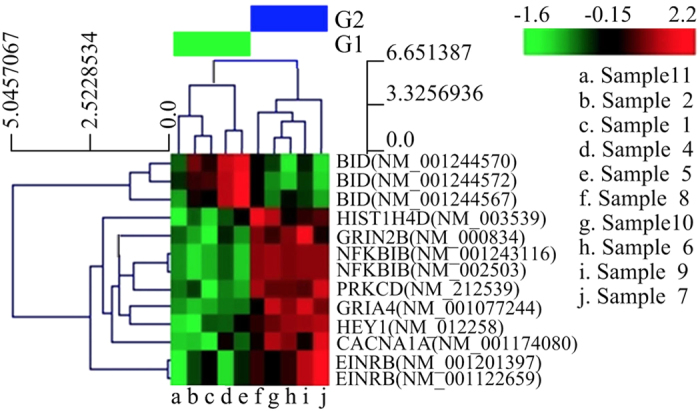
The heat map represents the cluster diagram of 9 GOIs in 10 samples. The differentiation ratio ranged from −1.6 to 2.2. Each row represents a gene, and each column represents a sample. There is clustering into two groups, corresponding to the grouping of NSTA patients and the control individuals. Red, hypermethylation; green, hypomethylation; black, no significant difference in methylation level. Note: G1, NSTA group; G2, control group. Sample 1 represents the anodontia female, and sample 6 represents her brother. Samples 2, 4, 5, and 11 refer to the four NSTA patients, while samples 7, 8, 9, and 10 refer to the four healthy controls.

The heat map for double-blind DEP cluster analysis is shown in Fig. 4. As can be seen from differences in genomic methylation level, statistically significant differences in methylation levels were observed between the two groups (*t* test, *P* < 0.05), which indicates that the methylation levels played a role in distinguishing between NSTA patients and control individuals. The differences in methylation levels of the 9 GOI in 10 samples showed that, a high methylation level was clustered in NFKBIB, PRKCD, CACNA1A, GRIA4, EDNRB, GRIN2B, HIST1H4D, and HEY1. A low methylation level was clustered in BID, in comparison with the control.

### GO and pathway analyses

**Figure 5:**
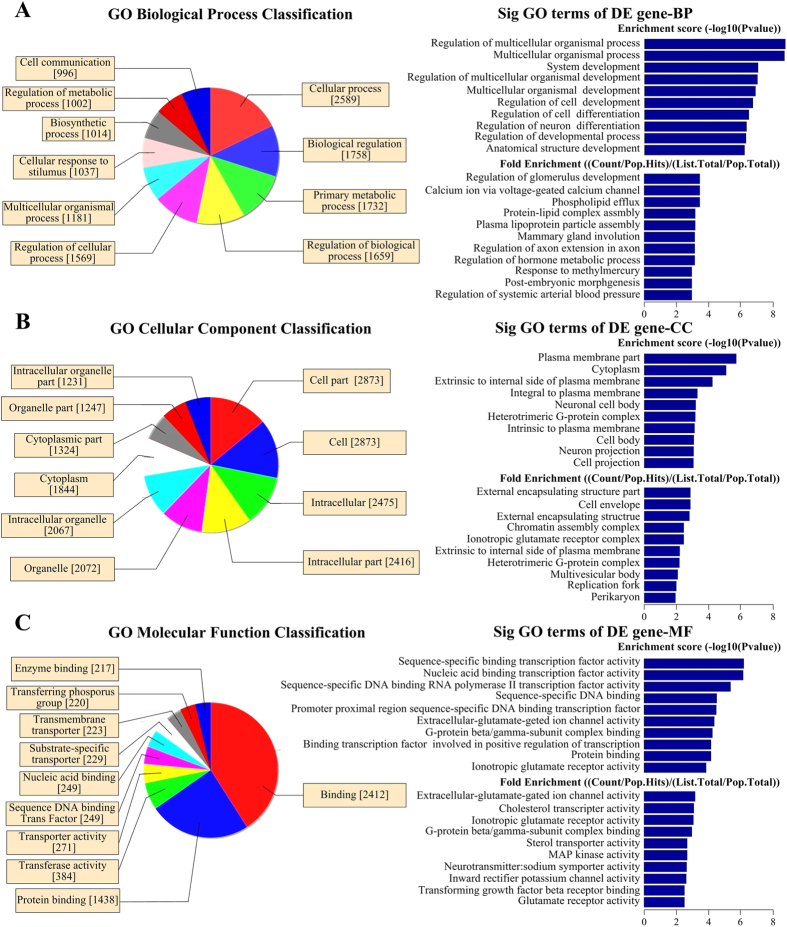
Bio-information analysis of differentially methylated genes. At left are pie diagrams of epidermal differentiation genes enriched for promoter methylation. At right are images depicting the results of gene ontology analysis of gene promoters enriched for DNA methylation in both groups. The x-axis represents the *P*-values for each gene ontology category shown that contains enrichment for DNA methylation (*P*-values < 5). **(A)** BP, Biological process. (**B**) CC, cellular component. (**C**) MF, Molecular function.

The 9 genes went through GO and pathway (DAVID) analysis. The results revealed these genes to be involved in various pathways that regulate multicellular processes and developmental processes, including chromatin structure, energy metabolism, cell signal transduction, protein transcription and synthesis, and cell apoptosis. The genes are described in Fig. 5.

## Discussion

Tooth development involves reciprocal actions between neural crest ectomesenchyme and ectodermal epithelium^1^,^37^, and the etiology of tooth agenesis results from an interaction among multiple genes and epigenetic and environmental factors. In the post-genomic era, epigenetic aspects have drawn much attention in recent years, and there is growing evidence revealing the critical role of epigenetic regulation in determining phenotypes.

To the best of our knowledge, this study was the first to analyze global differential methylation expression in tooth agenesis and to suggest that non-syndromic hypodontia/anodontia is related to simultaneous methylation level change of multiple gene promoters. We detected 6636 genes with different methylation levels between NSTA patients and unaffected control individuals. The results of DEP analysis (Fig. 3) showed that NSTA patients possessed a higher global methylation level than did the control individuals. Meanwhile, the PCA analysis demonstrated similar results based on the probe-specific analysis of MeD-chip. As with the significant contribution in PCA results, a group of genes presented evident differences in methylation status between the NSTA subjects and the control individuals. Through the bio-information analysis, these genes were scattered in a network of signaling pathways which appeared to have a close association in bio-function. We screened out 9 top differentially methylated genes of interest (Fig. 3D).

As shown in Appendix Figure 1 and 6, the 9 GOIs showed massive involvement in three signaling pathways in cartilage, bone, tooth, and neural development. They reticulated MARK, Notch, and Wnt/Ca^2+^ pathways. NFκBIB, with the highest difference in methylation, encoded an inhibitor of NFκB expression. NFκB plays an important role in tooth development, which is associated with tooth eruption in the dental follicle of the mouse^38^, and contributes to tooth number and shape^39^. Moreover, the activation of NFκB depends on PKC activation. The proteins encoded by PRKCD are multifunctional enzymes that bond the membrane receptors via the activation of G protein coupling systems and then trigger a series of intra-membrane activities^40^. PRKCD has been reported to mediate ameloblast differentiation and BMP-4-induced osteoblastic differentiation^41^. Hey 1 is an important downstream gene target in Notch signaling pathway, and the results indicated that the methylation state changes of Hey may be a subsequent reaction in Notch pathway activation. There is cross-talk between NFκB and HEY-1, since the expression of HEY-1 is suppressed when Ikk2 (a regulation protein of NFκB) expression is reduced^42^. CACNA1A encodes proteins involved in the Ca^2+^ channel and may influence the Wnt/Ca^2+^ pathway via Ca^2+^ level changes^43^. In addition, the CACN gene is closely related to the upstream modulation of Ras in the MAPK pathway, provided that RasGRF and RasGR are modulated by Ca^2+ ^44. BID is an apoptosis-related protein, involved in the NFκB pathway through IkB-alpha^45^. GRIA4 is the main excitatory neurotransmitter of mammals, related to the expression of CACNG2^46^ and PRKCG^47^.

Moreover, GRIN2B, CACNA1A, EDNRB, GRIA4, and BID are also reported to be associated with various human neurological disorders, such as migraine, epilepsy, Alzheimer’s disease, etc.^48^,^49^,^50^.

These GOIs are involved in important pathways in neural development/disease or in the significant pathways in tooth development, regulating multiple bio-activities including chromatin structure, intracellular calcium, mitochondrial function, cell signal transduction, protein transcription and synthesis, and cell apoptosis (Fig. 5). The close relationship of neural genes with dental development genes in the methylation state reflects their homogenous origin. This result was consistent with recent experimental discoveries^51^ and various tooth-neuropathy syndromes which indicate that the change in the methylation states of individual genes or gene sets may influence the development of both systems.

As with the classic “epigenetic-landscape” metaphor provided by Waddington^52^, we propose that these methylation changes may be a kind of reaction in responses to minor variations in the spatial and temporal expression of local signaling in odontogenic cells and, through some kind of amplification mechanism, lead to quite major differences in the final appearance of the dentition. Similarly, it has been presumed, from MZT pair studies^18^, that those twins who have the same genotypes but display different expressions in dentition may have a genetic predisposition that places them near the threshold for either missing or supranumerary teeth. Nevertheless, local epigenetic variations during the odontogenesis process determine on which side of the threshold they fall.

Many factors can influence global DNA methylation, both in the establishment of methylation and in the maintenance of methylation over time. Putative factors investigated to date have included age, ethnicity, nutrition, chemical exposures, and tobacco^15^,^16^,^17^,^18^. All of the NSTA patients had a normal phenotype except for 2 missing mandibular incisors and were otherwise free of any systemic diseases or abnormalities.

As for other frequently reported non-syndromic hypodontia-associated genes, like EDA, PAX, MSX, and AXN, there were no significant differences observed in the promoter methylation levels of these genes. Their association with hypodontia may function in other mechanisms, such as DNA sequence change or other CpG sites with methylation levels correlated with these gene sites may modify the interactions between DNA sequence and nuclear proteins, resulting in a change in gene expression. Further study on this mechanism is needed.

Our study suffers from a small sample size, due to the difficulties of obtaining phenotype- and gene-matched pairs. As validated in our previous study^14^, NSTA patients and control individuals, and the female proband and her younger brother share the same gene variants in PAX9, MSX1, AXIN2, and EDA, the genes most frequently reported to be related to hypodontia. Whole-genome sequencing could not be carried out due to financial constraints, so we cannot guarantee that the control individuals with normal phenotypes are not the carriers of other mutated genes. Thus, there is inevitable bias in this study. In particular, as reflected in the qPCR validation, the obvious within-group differences greatly compromised the reliability of the study results; therefore, a larger sample size is needed. However, since this was a pilot study, such bias should be tolerated since tentative qualitative testing was our primary goal, and we will perform more specific mechanism investigations in a further study (already in the initial stages) with a larger sample size. To further investigate methylation changes in tooth development, we plan to target downstream protein expression and signal pathways via silencing or up-/down-regulation of these genes of interest.

Conversely, epigenetic regulation is tissue-specific. In this study, we used the DNA from oral mucosal epithelial cells. It is plausible that, in only this tissue type, such change in the epigenetic profile is related to hypodontia. In this regard, the crucial epigenetic changes in fact might be more substantial than reported here. Future studies on epigenetic profiling of various cell types and the identification of cells specifically reflecting methylation in odontogenesis are warranted to gain a greater understanding of the epigenetic dysregulation in hypodontia.

## Conclusion

In conclusion, the use of MeD-chip and microarray analysis has highlighted numerous genes and pathways. Several genes with the most pronounced methylation changes between individuals with NSTA and control individuals have not been reported to play a direct role in non-syndromic tooth agenesis. Pathway analysis revealed that the alteration in methylation status affected a network of interconnected signaling pathways that included neurogenesis and tooth development. Although the precise nature of methylation influences is still unclear, they may be due to factors other than differences in methylation of DNA or acetylation of histones. We propose that they may reflect different responses of odontogenic cells to minor variations in the spatial and temporal expression of local signaling molecules passing between cells during tooth development. This hypothesis warrants further investigation.

## Methods

### Study population

The study samples were chosen from the previously reported study sample^14^. We recruited four patients with non-syndromic tooth agenesis (NSTA), congenitally missing two mandibular incisors, and four healthy people with normal dentition as control individuals, in addition to the two siblings. They were matched in terms of the allele and genotype frequencies of the important tooth-agenesis-related genes genes (MSX1, PAX9, AXIN2, and EDA).

All participants in the study were ethnic Han Chinese females between 20 and 30 years old.

Statement: All participants were fully informed of the purposes of the study and provided written consent. The research was approved by the Ethics Committee of Shanghai Tenth People’s Hospital (No: 2012-Res-033). The Helsinki Declaration was adhered to throughout the study.

Retrospective data were reviewed and panoramic radiographs were obtained to confirm the diagnosis of tooth agenesis for each participant, who also underwent a complete oral examination with a thorough clinical investigation of other tissues of ectodermal origin, including skin, hair, nails, sweat glands, ears, and eyes; the examinations were performed by an experienced doctor.

### DNA sample extraction and preparation

Genomic DNA (gDNA) samples were extracted from buccal epithelial cells by the Chelex-100 (Sigma) method^35^, and the purified gDNA was quantified and assessed by NanoDrop ND-1000. Genomic DNA of each sample was sonicated to ~200–1000 bp with a Bioruptor sonicator (Diagenode). The gDNA and each sheared DNA were quantified by Nanodrop ND-1000, then analyzed by agarose gel electrophoresis to ensure the integrity and check the size of sheared DNA, respectively, for the following experiment.

### Methylation DNA chip (MeD-chip)

Immunoprecipitation of methylated DNA was performed using Biomag^TM^ magnetic beads coupled with mouse monoclonal antibody against 5-methylcytidine. The immunoprecipitated DNA was eluted and purified by phenol chloroform extraction and ethanol precipitation. The MeDIP-enriched DNA was amplified with a WGA kit from Sigma-Aldrich (GenomePlex^®^ Complete Whole Genome Amplification [WGA2 kit]). The amplified DNA samples were then purified with a QIAquick PCR purification kit (Qiagen). The total input and immunoprecipitated DNA were labeled with Cy3- and Cy5-labeled random 9-mers, respectively, and hybridized to NimbleGen Human DNA Methylation 2.1 M Deluxe Promoter Arrays, which is a single array design that includes 27,867 CpG islands, all RefSeq gene promoter regions (from about −8 kb to +3 kb of the TSSs), and 730 miRNAs (from −15 kb to mature miRNA) totally covered by ~2.1 M probes. Scanning was performed with the Axon GenePix 4000B microarray scanner. The detailed protocol has been described in Palmke *et al*.^27^ and Hou *et al*.^36^.

### Data analysis

Raw data were extracted as pair files by NimbleScan software. To avoid technical variability and evaluate methylation differences between and among samples, the log2-ratio for arriving at a raw data value should be normalized. We performed median-centering, quantile normalization, and linear smoothing by the bioconductor packages Ringo, limma, and MEDME. After normalization, normalized log2-ratio data were created for each sample. From the normalized log2-ratio data, a sliding-window peak-finding algorithm, provided by NimbleScan v2.5 (Roche-NimbleGen), was applied to find the enriched peaks with specified parameters (sliding window width, 750 bp; mini-probes per peak, 2; *P*-value minimum cutoff, 2; maximum spacing between nearby probes within peak, 500 bp). The identified peaks were mapped to genomic features: transcripts, CpG islands, and miRNAs.

### Methylation enrichment and peak-finding

From the normalized log2-ratio data, a sliding-window (750 bp) peak-finding algorithm provided by NimbleScan v2.5 (Roche-NimbleGen) was applied for analysis of the MeDIP-chip data. A one-sided Kolmogorov-Smirnov (KS) test was applied to determine whether the probes were drawn from a significantly more positive distribution of intensity log2-ratios than those in the rest of the array. Each probe received a −log10 *P*-value score from the windowed KS test around that probe. If several adjacent probes rose significantly above a set threshold, the region was assigned to an enrichment peak (EP). NimbleScan detected peaks by searching for at least 2 probes above a *P*-value minimum cutoff (−log10) of 2. Peaks within 500 bp of each other were merged.

### Differential enrichment peaks (DEP) analysis by the M′ method

When comparing two groups’ differentially enriched regions, we averaged the log2-ratio values for each group (NSTA and Control) and calculated the M′ value (defined by the following equation) for each probe. Then we re-ran the NimbleScan sliding-window peak-finding algorithm on these data to find the DEP.

M′=Average(log2 MeDIP_E_/Input_E_) − Average(log2 MeDIP_C_/Input_C_)

The DEP called by the NimbleScan algorithm were filtered according to the following criteria:

- At least one of the two groups had a median (log2 MeDIP/Input) > = 0.3 and M′ > 0.
- At least half of the probes in a peak had a coefficient of variability (CV) < = 0.8 in both groups.

The identified DEP was mapped to genomic features: transcripts, CpG islands, and miRNAs.

### GO and pathway analysis

Functional analyses of hypermethylated genes were performed with the Database for Annotation, Visualization and Integrated Discovery (DAVID)^32^,^53^, which can search blocks of functionally related genes according to different criteria. This search was independent of methodological differences between the microarray analysis tools. A database of epithelially affiliated organ-development-related methylation sites and a database of epithelium-mesenchymal organ-development-related methylation sites were established, among which we focused on abnormal methylation sites.

### Statistical analysis

Quantitative data are shown as mean values ± SD. Statistical analyses were performed by the independent-samples *t* test. *P* < 0.05 was regarded as statistically significant.

## Additional Information

**How to cite this article**: Wang, J. *et al*. DNA methylation is critical for tooth agenesis: implications for sporadic non-syndromic anodontia and hypodontia. *Sci. Rep*. **6**, 19162; doi: 10.1038/srep19162 (2016).

## Supplementary Material

Supplementary Information
