# Supplementary material for: DNA methylation is critical for tooth agenesis: implications for sporadic non-syndromic anodontia and hypodontia
Source: Sci Rep. 2016 Jan 13;6:19162. doi: 10.1038/srep19162 (PMC4725352; doi:10.1038/srep19162)
Supplement: Supplementary Information [file srep19162-s1.doc]

**Title:** **DNA methylation is critical for tooth agenesis: implications for sporadic** **non-syndromic anodontia and hypodontia**

**Authors: Jing Wang, , Ke Sun, Jing Xie, Yun Shen, Yuanzhi Xua, Renhuan Huang, Yiming Zhang, Chenyuan Xu, Xu Zhang, Raorao Wang, Yunfeng Lin**

**Supplementary Figure 1:** flow chart

An intersection of both

**Subjects:** 5 NSTA patients and 5 controls

**Sample**: buccal epithelial cells

**DNA extraction Quality control**

gDNA sonication & denature

Immunoprecipitation & WGA

**Microarray hybridization ( MeDIP-chip)**

Correlation Matrix

MA-Plot

1.agarose gel electrophoresis

2.qPCR assessment for enrichment fold-enrichment

**Quality control**

**Data analysis**

Data normalization: Median-centering, quantile normalization and linear smoothing

**Probe-specific analysis:**

Probe with the fold change ratio >2 and < 0.05 (P< 0.05) were used as a cutoff for scoring high and low methylation level respectively. The genes in which at least one probe with differentiated methylation score were identified as candidate genes

NimbleScan v2.5 analysis**:**

1.Methylation Enrichment and Peak-Finding

2. DEP Analysis Using M’ Method

**9 genes:**

NFKBIB, PRKCD, CACNA1A, GRIA4, EIDDNRB,GRIN2B,

BID,HIST1H4D,HEY1

Inclusion criterion:

1. Peak score >2, P< 0.05

2.Dis. to TSS within -3kb to +3kb

Differentiated methylation: 177 genes

Hyper-methylation: 224 probes, 131genes

Hypo-methylation: 42 probes, 32 genes

Differentiated methylation: 266 probes, 163genes

**GO & pathway analysis**

**Supplementary Figure 2**

Comparison of the genome-wide enrichment peak distribution in promoter regions in chr1 of 10 samples. Differences in particular loci are noted with red circles.


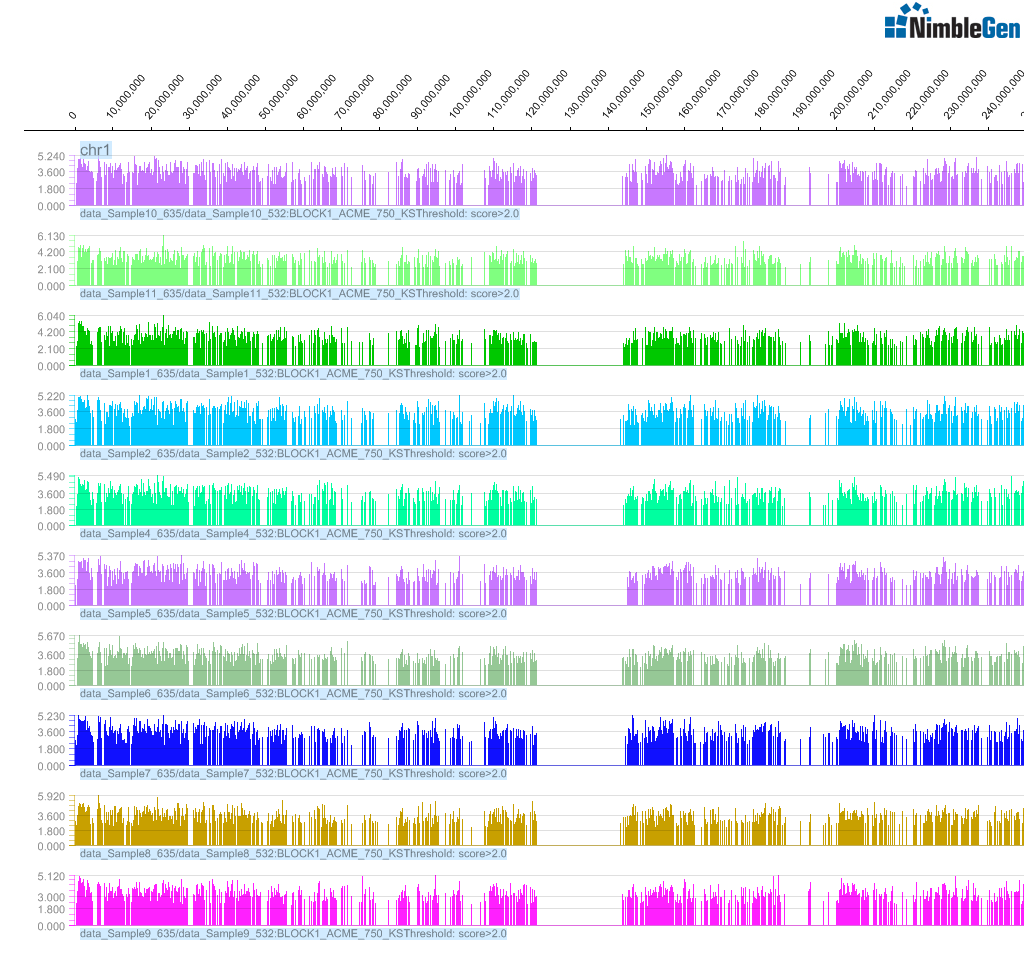


**Supplementary Table 1.** **The tabulated results of NimbleScan v2.5 analysis**

**(Inclusion criteria: [1] peak score > 2, P < 0.05; [2] distance to TSS within -3 kb to +3 kb).**

| **Gene Name** | **Peak Chromosome** | **Peak Score** | **Peak to TSS** | **Absolute value** |
| --- | --- | --- | --- | --- |
| NFKBIB | chr19 | 4.13 | -140 | **140** |
| NFKBIB | chr19 | 4.13 | -370 | **370** |
| PRKCD | chr3 | 4.04 | 1942 | **1942** |
| PPP1R1A | chr12 | 3.86 | -2741 | **2741** |
| NTF3 | chr12 | 3.8 | -1107 | **1107** |
| FGFRL1 | chr4 | 3.74 | 289 | **289** |
| FGFRL1 | chr4 | 3.74 | 781 | **781** |
| FGFRL1 | chr4 | 3.74 | 931 | **931** |
| GPSM1 | chr9 | 3.7 | -2753 | **2753** |
| HIST2H2AC | chr1 | 3.69 | 27 | **27** |
| HIST2H2BE | chr1 | 3.69 | -319 | **319** |
| HIST2H2AB | chr1 | 3.69 | 914 | **914** |
| PRKCG | chr19 | 3.65 | -1647 | **1647** |
| MAP2K2 | chr19 | 3.63 | 2173 | **2173** |
| ATP2A1 | chr16 | 3.57 | -888 | **888** |
| GNG5 | chr1 | 3.56 | 82 | **82** |
| RYR2 | chr1 | 3.45 | 613 | **613** |
| FAT4 | chr4 | 3.44 | -973 | **973** |
| JMJD7-PLA2G4B | chr15 | 3.42 | 1771 | **1771** |
| PPP1CC | chr12 | 3.41 | -1638 | **1638** |
| ADRB3 | chr8 | 3.39 | 0 | **0** |
| WNT9A | chr1 | 3.38 | 844 | **844** |
| GNAS | chr20 | 3.37 | 1057 | **1057** |
| MAPK13 | chr6 | 3.37 | -1651 | **1651** |
| STX1A | chr7 | 3.37 | 2671 | **2671** |
| DLX3 | chr17 | 3.34 | 1426 | **1426** |
| CACNA1A | chr19 | 3.34 | 1922 | **1922** |
| PLA2G3 | chr22 | 3.28 | -2213 | **2213** |
| OTX2 | chr14 | 3.28 | 2221 | **2221** |
| OTX2 | chr14 | 3.28 | -2617 | **2617** |
| GRIA4 | chr11 | 3.22 | 128 | **128** |
| GRIA4 | chr11 | 3.22 | 739 | **739** |
| GRIK4 | chr11 | 3.21 | 2304 | **2304** |
| WNT9A | chr1 | 3.21 | 2713 | **2713** |
| PIK3R3 | chr1 | 3.2 | 366 | **366** |
| KCNJ6 | chr21 | 3.2 | -391 | **391** |
| PIK3R3 | chr1 | 3.2 | 694 | **694** |
| P2RX1 | chr17 | 3.2 | -2039 | **2039** |
| CALY | chr10 | 3.19 | -2639 | **2639** |
| CCKAR | chr4 | 3.18 | -2999 | **2999** |
| CALML3 | chr10 | 3.16 | -1125 | **1125** |
| H2AFY | chr5 | 3.13 | -85 | **85** |
| H2AFY | chr5 | 3.13 | 564 | **564** |
| GNAL | chr18 | 3.11 | -1213 | **1213** |
| GRM6 | chr5 | 3.1 | 2754 | **2754** |
| GNAS | chr20 | 3.07 | 631 | **631** |
| PPP1CA | chr11 | 3.07 | 2686 | **2686** |
| GRIN1 | chr9 | 3.06 | -499 | **499** |
| GAB1 | chr4 | 3.04 | -391 | **391** |
| HIST1H2AM | chr6 | 3.03 | 321 | **321** |
| HIST1H2BO | chr6 | 3.03 | -560 | **560** |
| HIST1H3J | chr6 | 3.03 | -2071 | **2071** |
| WNT5A | chr3 | 3.02 | -1911 | **1911** |
| OTX2 | chr14 | 3.01 | 1487 | **1487** |
| EDNRB | chr13 | 2.99 | -161 | **161** |
| EDNRB | chr13 | 2.99 | 775 | **775** |
| DDC | chr7 | 2.98 | 2032 | **2032** |
| NOS3 | chr7 | 2.97 | 2042 | **2042** |
| SHC2 | chr19 | 2.97 | 2258 | **2258** |
| GRIN2B | chr12 | 2.95 | -577 | **577** |
| WNT7B | chr22 | 2.94 | 1933 | **1933** |
| SH2B1 | chr16 | 2.9 | 10 | **10** |
| SH2B1 | chr16 | 2.9 | 28 | **28** |
| SH2B1 | chr16 | 2.9 | 246 | **246** |
| PRKACB | chr1 | 2.9 | 529 | **529** |
| BID | chr22 | 2.9 | 2406 | **2406** |
| BID | chr22 | 2.9 | 2859 | **2859** |
| GAS1 | chr9 | 2.89 | 219 | **219** |
| MAPK11 | chr22 | 2.89 | 2227 | **2227** |
| P2RX1 | chr17 | 2.88 | -164 | **164** |
| H2AFY2 | chr10 | 2.87 | 1090 | **1090** |
| HDAC3 | chr5 | 2.87 | -1355 | **1355** |
| HIST1H4D | chr6 | 2.86 | -93 | **93** |
| HEY1 | chr8 | 2.86 | -2009 | **2009** |
| SMAD3 | chr15 | 2.85 | 508 | **508** |
| CCS | chr11 | 2.84 | 71 | **71** |
| CAT | chr11 | 2.84 | -158 | **158** |
| BST1 | chr4 | 2.84 | 1330 | **1330** |
| ADCY8 | chr8 | 2.84 | -1539 | **1539** |
| HDAC5 | chr17 | 2.84 | 2026 | **2026** |
| KCNJ5 | chr11 | 2.83 | 2578 | **2578** |
| ORAI3 | chr16 | 2.82 | 92 | **92** |
| ZNF274 | chr19 | 2.82 | 151 | **151** |
| PPP1CA | chr11 | 2.82 | 1485 | **1485** |
| SMAD3 | chr15 | 2.81 | -510 | **510** |
| TP53 | chr17 | 2.81 | 1416 | **1416** |
| CACNA1C | chr12 | 2.81 | 1932 | **1932** |
| NTF4 | chr19 | 2.79 | 111 | **111** |
| MAPK12 | chr22 | 2.79 | -564 | **564** |
| HOMER2 | chr15 | 2.78 | -111 | **111** |
| FGF3 | chr11 | 2.78 | 263 | **263** |
| KLF10 | chr8 | 2.78 | -1686 | **1686** |
| MAP2K3 | chr17 | 2.77 | -336 | **336** |
| SHC1 | chr1 | 2.75 | 30 | **30** |
| CHRNA7 | chr15 | 2.75 | 452 | **452** |
| GRIN3A | chr9 | 2.74 | 1588 | **1588** |
| P2RX2 | chr12 | 2.74 | 1877 | **1877** |
| JMJD7-PLA2G4B | chr15 | 2.74 | -2822 | **2822** |
| ADCY8 | chr8 | 2.73 | 148 | **148** |
| ATF4 | chr22 | 2.73 | -732 | **732** |
| ADCY2 | chr5 | 2.71 | -189 | **189** |
| ORAI1 | chr12 | 2.7 | 2192 | **2192** |
| PTHLH | chr12 | 2.68 | -129 | **129** |
| PTHLH | chr12 | 2.68 | 1892 | **1892** |
| NOS1 | chr12 | 2.67 | -1910 | **1910** |
| BOC | chr3 | 2.65 | 132 | **132** |
| PPP2R5B | chr11 | 2.64 | -1179 | **1179** |
| GNB5 | chr15 | 2.64 | 1874 | **1874** |
| CASP3 | chr4 | 2.61 | -163 | **163** |
| IHH | chr2 | 2.61 | 1257 | **1257** |
| NOS3 | chr7 | 2.61 | -1322 | **1322** |
| NOS3 | chr7 | 2.61 | 1425 | **1425** |
| FGF17 | chr8 | 2.6 | 124 | **124** |
| TOMM40 | chr19 | 2.6 | -382 | **382** |
| CEBPB | chr20 | 2.6 | 1511 | **1511** |
| NOTCH1 | chr9 | 2.59 | 217 | **217** |
| RPS6KA1 | chr1 | 2.59 | 558 | **558** |
| WNT3 | chr17 | 2.59 | 2710 | **2710** |
| KLK4 | chr19 | 2.58 | -1193 | **1193** |
| ARC | chr8 | 2.57 | -383 | **383** |
| SLC38A1 | chr12 | 2.56 | 1111 | **1111** |
| NGF | chr1 | 2.55 | 760 | **760** |
| GRIA2 | chr4 | 2.55 | 1959 | **1959** |
| GRIA2 | chr4 | 2.55 | 2043 | **2043** |
| GRIN2A | chr16 | 2.54 | -166 | **166** |
| KCNJ12 | chr17 | 2.54 | 290 | **290** |
| GRIN2A | chr16 | 2.54 | -514 | **514** |
| GRIN2A | chr16 | 2.54 | -853 | **853** |
| VDAC1 | chr5 | 2.51 | -383 | **383** |
| VDAC1 | chr5 | 2.51 | -550 | **550** |
| SLC8A2 | chr19 | 2.51 | 895 | **895** |
| HIST1H3G | chr6 | 2.5 | 191 | **191** |
| GNAO1 | chr16 | 2.5 | 280 | **280** |
| SLC6A3 | chr5 | 2.5 | -793 | **793** |
| GRIN2D | chr19 | 2.5 | -1374 | **1374** |
| HIST1H2BI | chr6 | 2.5 | -1782 | **1782** |
| CHRNB4 | chr15 | 2.48 | 415 | **415** |
| PTGER3 | chr1 | 2.48 | 535 | **535** |
| SMAD3 | chr15 | 2.48 | 1761 | **1761** |
| KLF10 | chr8 | 2.47 | 192 | **192** |
| KLF10 | chr8 | 2.47 | -1622 | **1622** |
| IL10 | chr1 | 2.45 | 2415 | **2415** |
| SMAD6 | chr15 | 2.44 | -694 | **694** |
| GNG10 | chr9 | 2.44 | 1948 | **1948** |
| PPP2R5D | chr6 | 2.43 | 54 | **54** |
| SLC6A3 | chr5 | 2.42 | 2562 | **2562** |
| CHP2 | chr16 | 2.4 | -509 | **509** |
| RPS6KA4 | chr11 | 2.4 | 2018 | **2018** |
| CREB5 | chr7 | 2.4 | -2426 | **2426** |
| RPS6KA2 | chr6 | 2.39 | -19 | **19** |
| PTCH2 | chr1 | 2.39 | -133 | **133** |
| KRAS | chr12 | 2.36 | -52 | **52** |
| CHRNA4 | chr20 | 2.34 | 226 | **226** |
| SERPINE1 | chr7 | 2.34 | -237 | **237** |
| PIK3R5 | chr17 | 2.34 | -276 | **276** |
| GNG7 | chr19 | 2.34 | 340 | **340** |
| MYLK | chr3 | 2.34 | 1768 | **1768** |
| FGF19 | chr11 | 2.34 | 1967 | **1967** |
| MAPK7 | chr17 | 2.34 | 2468 | **2468** |
| PDGFRB | chr5 | 2.33 | 73 | **73** |
| DRD1 | chr5 | 2.33 | 181 | **181** |
| MYOD1 | chr11 | 2.33 | 413 | **413** |
| PPP1CB | chr2 | 2.33 | 442 | **442** |
| PPP1CB | chr2 | 2.33 | 454 | **454** |
| WNT7B | chr22 | 2.33 | -2395 | **2395** |
| ADRBK1 | chr11 | 2.33 | 2817 | **2817** |
| NFKBIE | chr6 | 2.32 | 1238 | **1238** |
| BAX | chr19 | 2.31 | 677 | **677** |
| WNT4 | chr1 | 2.31 | -773 | **773** |
| MAPK11 | chr22 | 2.31 | -2123 | **2123** |
| MAOB | chrX | 2.3 | 1359 | **1359** |
| FGFR3 | chr4 | 2.3 | 2799 | **2799** |
| GNAI2 | chr3 | 2.3 | -2867 | **2867** |
| STIM1 | chr11 | 2.29 | 607 | **607** |
| GREM1 | chr15 | 2.29 | 1542 | **1542** |
| PDE1C | chr7 | 2.27 | -184 | **184** |
| MAPK14 | chr6 | 2.27 | 829 | **829** |
| PLCB2 | chr15 | 2.27 | 939 | **939** |
| GRIN2C | chr17 | 2.27 | -1383 | **1383** |
| FGF17 | chr8 | 2.27 | -2328 | **2328** |
| DISP2 | chr15 | 2.26 | 948 | **948** |
| CALML6 | chr1 | 2.26 | 1399 | **1399** |
| BAD | chr11 | 2.25 | -101 | **101** |
| BMP7 | chr20 | 2.25 | 1174 | **1174** |
| KCNQ5 | chr6 | 2.24 | -131 | **131** |
| WNT10A | chr2 | 2.24 | -295 | **295** |
| IRAK1 | chrX | 2.24 | 2680 | **2680** |
| GNG3 | chr11 | 2.23 | -430 | **430** |
| CHAT | chr10 | 2.22 | 925 | **925** |
| WNT9A | chr1 | 2.22 | -1820 | **1820** |
| CHAT | chr10 | 2.22 | 1854 | **1854** |
| GNA15 | chr19 | 2.21 | 59 | **59** |
| ITPKC | chr19 | 2.21 | 871 | **871** |
| SH2B2 | chr7 | 2.2 | 504 | **504** |
| PTPN11 | chr12 | 2.18 | 439 | **439** |
| ARC | chr8 | 2.18 | -1528 | **1528** |
| H2AFX | chr11 | 2.17 | 1098 | **1098** |
| CAMK4 | chr5 | 2.16 | 210 | **210** |
| IFRD1 | chr7 | 2.16 | -385 | **385** |
| DRD2 | chr11 | 2.16 | -399 | **399** |
| IFRD1 | chr7 | 2.16 | -2015 | **2015** |
| SPHK1 | chr17 | 2.15 | 1180 | **1180** |
| SPHK1 | chr17 | 2.15 | 1779 | **1779** |
| H2AFB2 | chrX | 2.15 | 1913 | **1913** |
| H2AFB1 | chrX | 2.15 | 1921 | **1921** |
| H2AFB3 | chrX | 2.15 | 1921 | **1921** |
| PLCD1 | chr3 | 2.14 | -512 | **512** |
| FGF20 | chr8 | 2.1 | 421 | **421** |
| ADCY9 | chr16 | 2.09 | 1952 | **1952** |
| PIK3R5 | chr17 | 2.08 | 766 | **766** |
| PIK3R5 | chr17 | 2.08 | -1535 | **1535** |
| GSK3A | chr19 | 2.07 | -678 | **678** |
| FOS | chr14 | 2.07 | 2573 | **2573** |
| TGFB2 | chr1 | 2.06 | 715 | **715** |
| NTF3 | chr12 | 2.05 | -565 | **565** |
| CHRM1 | chr11 | 2.05 | 1393 | **1393** |
| MYOD1 | chr11 | 2.05 | 1912 | **1912** |
| HIST1H3F | chr6 | 2.04 | 172 | **172** |
| HIST1H2BH | chr6 | 2.04 | -1215 | **1215** |
| GRIK5 | chr19 | 2.02 | -652 | **652** |

**Supplementary Table 2.** The tabulated results of probe-specific analysis.

| **Gene Info.** | **Probe Info.** | **Fold Change and *P*-value** | |
| --- | --- | --- | --- |
| **Gene Name** | **Chromosome** | **Fold Change** | ***P*-value** |
| GET4 | chr7 | 0.291710271 | 0.002927122 |
| CRLF2 | chrY | 0.309305827 | 0.007371929 |
| CRLF2 | chrY | 0.309305827 | 0.007371929 |
| LBH | chr2 | 0.322813401 | 0.028524576 |
| CEP95 | chr17 | 0.339977686 | 0.000558122 |
| MIR3064 | chr17 | 0.339977686 | 0.000558122 |
| DDX5 | chr17 | 0.339977686 | 0.000558122 |
| MIR5047 | chr17 | 0.339977686 | 0.000558122 |
| MIR1247 | chr14 | 0.342151292 | 9.10E-05 |
| GALNT7 | chr4 | 0.34223826 | 0.043382931 |
| VAV1 | chr19 | 0.366758457 | 0.000489792 |
| SH2D3A | chr19 | 0.366758457 | 0.000489792 |
| GLP1R | chr6 | 0.367768457 | 0.041093643 |
| AADAC | chr3 | 0.368658788 | 0.006040266 |
| FAM99A | chr11 | 0.369417002 | 0.000430653 |
| IFITM3 | chr11 | 0.369792029 | 8.75E-06 |
| ASMT | chrX | 0.370322286 | 0.002337868 |
| SLC8A3 | chr14 | 0.374197803 | 0.000845478 |
| NFKBIB | chr19 | 0.374939251 | 4.19E-06 |
| SIRT2 | chr19 | 0.374939251 | 4.19E-06 |
| SIRT2 | chr19 | 0.374939251 | 4.19E-06 |
| NFKBIB | chr19 | 0.374939251 | 4.19E-06 |
| SIRT2 | chr19 | 0.374939251 | 4.19E-06 |
| MYH7 | chr14 | 0.376734852 | 0.001847976 |
| RAB3B | chr1 | 0.384530063 | 0.005071731 |
| NFKBIB | chr19 | 0.38842712 | 0.00042374 |
| SIRT2 | chr19 | 0.38842712 | 0.00042374 |
| SIRT2 | chr19 | 0.38842712 | 0.00042374 |
| NFKBIB | chr19 | 0.38842712 | 0.00042374 |
| SIRT2 | chr19 | 0.38842712 | 0.00042374 |
| CRLF2 | chrY | 0.393003907 | 0.000916675 |
| CRLF2 | chrY | 0.393003907 | 0.000916675 |
| LOC728752 | chr19 | 0.394598228 | 0.001485296 |
| ZNF566 | chr19 | 0.394598228 | 0.001485296 |
| ZNF566 | chr19 | 0.394598228 | 0.001485296 |
| CD248 | chr11 | 0.397005265 | 0.01293283 |
| NSDHL | chrX | 0.399998721 | 0.002545097 |
| CETN2 | chrX | 0.399998721 | 0.002545097 |
| RPL36AL | chr14 | 0.400000101 | 0.00016416 |
| MGAT2 | chr14 | 0.400000101 | 0.00016416 |
| TIRAP | chr11 | 0.40222169 | 0.000949004 |
| LTBP4 | chr19 | 0.403688947 | 3.97E-06 |
| LTBP4 | chr19 | 0.403688947 | 3.97E-06 |
| LOC84989 | chr10 | 0.404735083 | 9.39E-05 |
| JMJD1C | chr10 | 0.404735083 | 9.39E-05 |
| CEP95 | chr17 | 0.405875887 | 1.44E-05 |
| MIR3064 | chr17 | 0.405875887 | 1.44E-05 |
| DDX5 | chr17 | 0.405875887 | 1.44E-05 |
| MIR5047 | chr17 | 0.405875887 | 1.44E-05 |
| LOC100134713 | chr7 | 0.408669514 | 0.000671805 |
| NDUFB2 | chr7 | 0.408669514 | 0.000671805 |
| CCDC155 | chr19 | 0.408949714 | 2.72E-05 |
| MIR1249 | chr22 | 0.411716622 | 0.000150183 |
| BARHL1 | chr9 | 0.41314014 | 0.000140356 |
| RAB3B | chr1 | 0.414664666 | 0.00159145 |
| GRHL3 | chr1 | 0.416077585 | 0.001354126 |
| GRHL3 | chr1 | 0.416077585 | 0.001354126 |
| GRHL3 | chr1 | 0.416077585 | 0.001354126 |
| PRKCD | chr3 | 0.416367528 | 0.000122081 |
| SDCCAG3 | chr9 | 0.41769376 | 0.000114654 |
| PMPCA | chr9 | 0.41769376 | 0.000114654 |
| MIR1249 | chr22 | 0.418717665 | 0.000977779 |
| ALOX15B | chr17 | 0.424149259 | 0.000321992 |
| RRP7A | chr22 | 0.425783949 | 0.009039331 |
| RPL36AL | chr14 | 0.430416423 | 0.000482551 |
| MGAT2 | chr14 | 0.430416423 | 0.000482551 |
| C16orf42 | chr16 | 0.430638086 | 0.008858725 |
| GNPTG | chr16 | 0.430638086 | 0.008858725 |
| GRIA4 | chr11 | 0.433345884 | 0.000299934 |
| GRIA4 | chr11 | 0.433345884 | 0.000299934 |
| ALOX15B | chr17 | 0.436355604 | 0.011902011 |
| PEMT | chr17 | 0.439450121 | 0.000124142 |
| PEMT | chr17 | 0.439995318 | 0.006021779 |
| LDB1 | chr10 | 0.440468141 | 0.045754586 |
| LDB1 | chr10 | 0.440468141 | 0.045754586 |
| ARNT2 | chr15 | 0.440474205 | 0.012532859 |
| VAMP7 | chrX | 0.441107257 | 0.002734077 |
| NUP43 | chr6 | 0.441286691 | 0.000175198 |
| PCMT1 | chr6 | 0.441286691 | 0.000175198 |
| NIN | chr14 | 0.441803188 | 0.009289753 |
| SLC8A3 | chr14 | 0.443129229 | 8.49E-05 |
| LOC100134713 | chr7 | 0.443501872 | 7.21E-06 |
| NDUFB2 | chr7 | 0.443501872 | 7.21E-06 |
| IFITM3 | chr11 | 0.443904055 | 6.63E-05 |
| ATP6V0A1 | chr17 | 0.444474512 | 0.001520285 |
| LENG9 | chr19 | 0.44541504 | 0.010304661 |
| MOB3A | chr19 | 0.446419296 | 0.000899312 |
| KLHL24 | chr3 | 0.447634753 | 0.009891636 |
| NFKBIB | chr19 | 0.4483986 | 9.54E-05 |
| SIRT2 | chr19 | 0.4483986 | 9.54E-05 |
| SIRT2 | chr19 | 0.4483986 | 9.54E-05 |
| NFKBIB | chr19 | 0.4483986 | 9.54E-05 |
| SIRT2 | chr19 | 0.4483986 | 9.54E-05 |
| ECE1 | chr1 | 0.44911874 | 0.000414579 |
| ISLR | chr15 | 0.451496422 | 0.002791938 |
| ISLR | chr15 | 0.451496422 | 0.002791938 |
| LSM7 | chr19 | 0.451508253 | 0.014688553 |
| PRKCD | chr3 | 0.455095244 | 0.000369962 |
| PDCD4 | chr10 | 0.456798767 | 0.000596561 |
| LOC282997 | chr10 | 0.456798767 | 0.000596561 |
| HERC2P4 | chr16 | 0.457060908 | 0.020365066 |
| LOC728752 | chr19 | 0.457341443 | 0.000464509 |
| ZNF566 | chr19 | 0.457341443 | 0.000464509 |
| ZNF566 | chr19 | 0.457341443 | 0.000464509 |
| KCNS2 | chr8 | 0.458366109 | 0.042225604 |
| KCP | chr7 | 0.459327019 | 0.000705236 |
| TDRKH | chr1 | 0.459821734 | 0.001964821 |
| TCTE1 | chr6 | 0.460854143 | 0.000387482 |
| MIR1249 | chr22 | 0.461775946 | 0.001790193 |
| GPR146 | chr7 | 0.462367043 | 2.50E-05 |
| MIR202 | chr10 | 0.462658707 | 0.00269403 |
| GNG8 | chr19 | 0.463623474 | 0.00077138 |
| KIAA0232 | chr4 | 0.463697756 | 6.24E-05 |
| LSP1 | chr11 | 0.464046174 | 0.000308372 |
| LSP1 | chr11 | 0.464046174 | 0.000308372 |
| ARL5B | chr10 | 0.46404738 | 0.012814331 |
| LOC286238 | chr9 | 0.464590529 | 0.003041898 |
| NUDT18 | chr8 | 0.464902416 | 0.003905576 |
| MYH7 | chr14 | 0.46631986 | 0.000715796 |
| PDLIM3 | chr4 | 0.466835957 | 0.009134385 |
| MIR548I3 | chr8 | 0.46704404 | 0.001054721 |
| NDUFA4L2 | chr12 | 0.467575991 | 0.001946746 |
| CD248 | chr11 | 0.468939426 | 0.004994994 |
| LSM7 | chr19 | 0.470063034 | 0.001344498 |
| LSP1 | chr11 | 0.470379026 | 0.001125812 |
| LSP1 | chr11 | 0.470379026 | 0.001125812 |
| CEP95 | chr17 | 0.470626314 | 6.27E-05 |
| MIR3064 | chr17 | 0.470626314 | 6.27E-05 |
| DDX5 | chr17 | 0.470626314 | 6.27E-05 |
| MIR5047 | chr17 | 0.470626314 | 6.27E-05 |
| FAM99A | chr11 | 0.470852796 | 0.000514109 |
| MYH7 | chr14 | 0.471507989 | 0.004615431 |
| LOC113230 | chr19 | 0.472689694 | 0.001775789 |
| C16orf45 | chr16 | 0.473208758 | 0.000177787 |
| LOXL4 | chr10 | 0.47389515 | 0.015069746 |
| TMEM88 | chr17 | 0.474487872 | 0.016007938 |
| LSMD1 | chr17 | 0.474487872 | 0.016007938 |
| CYB5D1 | chr17 | 0.474487872 | 0.016007938 |
| GAB2 | chr11 | 0.474516188 | 0.000815854 |
| RAB3B | chr1 | 0.475397146 | 0.007814975 |
| CASP2 | chr7 | 0.475539784 | 0.000704733 |
| TMEM139 | chr7 | 0.475539784 | 0.000704733 |
| CASP2 | chr7 | 0.475539784 | 0.000704733 |
| TBC1D9 | chr4 | 0.478302569 | 0.00176701 |
| CACNA1A | chr19 | 0.478817364 | 0.001032023 |
| ABCA7 | chr19 | 0.478971109 | 0.01579098 |
| FBP1 | chr9 | 0.479233396 | 7.55E-06 |
| PLEKHM1P | chr17 | 0.480190327 | 6.01E-05 |
| ALG8 | chr11 | 0.480353634 | 0.002292979 |
| GRIA4 | chr11 | 0.480464152 | 0.018826039 |
| GRIA4 | chr11 | 0.480464152 | 0.018826039 |
| CAMSAP1 | chr9 | 0.480478411 | 0.013111787 |
| ATP6V0A1 | chr17 | 0.480625116 | 0.012765434 |
| SSBP4 | chr19 | 0.480717524 | 0.004331167 |
| ATP6V0A1 | chr17 | 0.48101635 | 0.014165991 |
| LSP1 | chr11 | 0.485747594 | 3.24E-05 |
| LSP1 | chr11 | 0.485747594 | 3.24E-05 |
| OSCP1 | chr1 | 0.485947508 | 0.010632536 |
| ZNF775 | chr7 | 0.486131392 | 0.026000339 |
| PCDH10 | chr4 | 0.487185447 | 0.00181292 |
| TGIF2 | chr20 | 0.487216303 | 0.021465155 |
| TGIF2 | chr20 | 0.487216303 | 0.021465155 |
| TGIF2-C20ORF24 | chr20 | 0.487216303 | 0.021465155 |
| TGIF2 | chr20 | 0.487216303 | 0.021465155 |
| EBF2 | chr8 | 0.487219048 | 0.012335055 |
| SLC16A3 | chr17 | 0.487304849 | 0.000282039 |
| SLC16A3 | chr17 | 0.487304849 | 0.000282039 |
| ALOX15B | chr17 | 0.487548569 | 0.000459031 |
| MIR548I3 | chr8 | 0.487571297 | 0.000456376 |
| SLC22A7 | chr6 | 0.487959139 | 0.001749563 |
| GPR63 | chr6 | 0.487971238 | 0.006841649 |
| NBL1 | chr1 | 0.489038073 | 0.011571686 |
| NBL1 | chr1 | 0.489038073 | 0.011571686 |
| NBL1 | chr1 | 0.489038073 | 0.011571686 |
| NBL1 | chr1 | 0.489038073 | 0.011571686 |
| SEMA6B | chr19 | 0.490441464 | 0.000102012 |
| PDZD7 | chr10 | 0.491275399 | 0.023426975 |
| TGIF2 | chr20 | 0.491482153 | 0.014638738 |
| TGIF2 | chr20 | 0.491482153 | 0.014638738 |
| TGIF2-C20ORF24 | chr20 | 0.491482153 | 0.014638738 |
| TGIF2 | chr20 | 0.491482153 | 0.014638738 |
| GAB2 | chr11 | 0.491618222 | 0.001100195 |
| HIST1H4D | chr6 | 0.49271298 | 0.000632957 |
| PARP14 | chr3 | 0.492790863 | 0.001148843 |
| LINC00338 | chr17 | 0.492794748 | 0.005577841 |
| SEC14L1 | chr17 | 0.492794748 | 0.005577841 |
| SEC14L1 | chr17 | 0.492794748 | 0.005577841 |
| SCARNA16 | chr17 | 0.492794748 | 0.005577841 |
| MTERFD2 | chr2 | 0.49285159 | 0.001484646 |
| NFKBIB | chr19 | 0.492860582 | 0.000574524 |
| SIRT2 | chr19 | 0.492860582 | 0.000574524 |
| SIRT2 | chr19 | 0.492860582 | 0.000574524 |
| NFKBIB | chr19 | 0.492860582 | 0.000574524 |
| SIRT2 | chr19 | 0.492860582 | 0.000574524 |
| KLHL7 | chr7 | 0.49336595 | 0.000398601 |
| KLHL7 | chr7 | 0.49336595 | 0.000398601 |
| GNG8 | chr19 | 0.494166484 | 0.000703474 |
| MRPL17 | chr11 | 0.494639407 | 0.00010837 |
| DNAJC19 | chr3 | 0.4950164 | 0.006944121 |
| DNAJC19 | chr3 | 0.4950164 | 0.006944121 |
| PLXNA4 | chr7 | 0.495395527 | 0.002220221 |
| RENBP | chrX | 0.495732598 | 0.029060246 |
| RAB3B | chr1 | 0.496000977 | 0.015731024 |
| AQP6 | chr12 | 0.496295641 | 0.000389454 |
| GRIN2B | chr12 | 0.496358814 | 0.00549945 |
| DNAJB8 | chr3 | 0.49648148 | 0.001900817 |
| ARHGEF15 | chr17 | 0.497080345 | 0.003769018 |
| ARHGEF15 | chr17 | 0.497080345 | 0.003769018 |
| C10orf93 | chr10 | 0.497181033 | 0.001565523 |
| RRP7A | chr22 | 0.49728514 | 0.010440601 |
| CEP95 | chr17 | 0.49746821 | 0.023912876 |
| MIR3064 | chr17 | 0.49746821 | 0.023912876 |
| DDX5 | chr17 | 0.49746821 | 0.023912876 |
| MIR5047 | chr17 | 0.49746821 | 0.023912876 |
| MOB3A | chr19 | 0.497723889 | 0.014218513 |
| ANKRD28 | chr3 | 0.498091186 | 0.013162761 |
| GRHL3 | chr1 | 0.498480863 | 0.01315508 |
| GRHL3 | chr1 | 0.498480863 | 0.01315508 |
| GRHL3 | chr1 | 0.498480863 | 0.01315508 |
| PYY2 | chr17 | 0.498538573 | 0.000183578 |
| LSM7 | chr19 | 0.498616073 | 0.007022952 |
| GUCA2A | chr1 | 0.498930946 | 0.001842375 |
| GDPD5 | chr11 | 0.499172133 | 0.003911189 |
| EDNRB | chr13 | 0.49972382 | 0.009566985 |
| EDNRB | chr13 | 0.49972382 | 0.009566985 |
| AIF1L | chr9 | 2.000448154 | 0.004625534 |
| SPOCK1 | chr5 | 2.000578629 | 0.000379258 |
| FAM155A | chr13 | 2.010071476 | 0.016365371 |
| LOC100505806 | chr5 | 2.017721446 | 0.034321446 |
| SEMA5A | chr5 | 2.017721446 | 0.034321446 |
| SNORD123 | chr5 | 2.017721446 | 0.034321446 |
| LRRC24 | chr8 | 2.019248369 | 0.035225253 |
| C8orf82 | chr8 | 2.019248369 | 0.035225253 |
| MSC | chr8 | 2.026706503 | 0.023645774 |
| CCDC116 | chr22 | 2.036298076 | 4.49E-05 |
| ABCG4 | chr11 | 2.042856019 | 0.015025988 |
| ABCG4 | chr11 | 2.042856019 | 0.015025988 |
| UBL4A | chrX | 2.043833627 | 0.002424426 |
| POLD3 | chr11 | 2.064005398 | 0.045177273 |
| LPPR2 | chr19 | 2.06650987 | 0.011106502 |
| LYRM1 | chr16 | 2.079983986 | 0.029210865 |
| LYRM1 | chr16 | 2.079983986 | 0.029210865 |
| FAM65A | chr16 | 2.102604465 | 0.005782041 |
| FAM65A | chr16 | 2.102604465 | 0.005782041 |
| FAM65A | chr16 | 2.102604465 | 0.005782041 |
| FAM65A | chr16 | 2.102604465 | 0.005782041 |
| MIR3198-1 | chr22 | 2.103800589 | 0.020593324 |
| BID | chr22 | 2.103800589 | 0.020593324 |
| BID | chr22 | 2.103800589 | 0.020593324 |
| MIR650 | chr22 | 2.106872748 | 0.00942808 |
| SDF4 | chr1 | 2.11974175 | 0.046466826 |
| OPN4 | chr10 | 2.177685984 | 0.027844767 |
| ZNF195 | chr11 | 2.215259463 | 0.013971839 |
| LOC650368 | chr11 | 2.215259463 | 0.013971839 |
| CABP2 | chr11 | 2.22083447 | 0.008189296 |
| BRP44L | chr6 | 2.276736854 | 0.018947018 |
| APOBEC3H | chr22 | 2.292983421 | 0.007241556 |
| HCFC1R1 | chr16 | 2.310053511 | 0.02464697 |
| LTC4S | chr5 | 2.311110912 | 0.029973825 |
| LCE1F | chr1 | 2.347815626 | 0.034812559 |
| C11orf82 | chr11 | 2.614397973 | 0.042141068 |
| AIF1L | chr9 | 2.743359689 | 0.028666675 |
| PDZD4 | chrX | 3.317215763 | 0.008084962 |
| UBL4A | chrX | 3.654119098 | 0.004904639 |
